# The Use of Dietary Supplements and Amino Acid Restriction Interventions to Reduce Frailty in Pre-Clinical Models

**DOI:** 10.3390/nu14142806

**Published:** 2022-07-08

**Authors:** Elise S. Bisset, Susan E. Howlett

**Affiliations:** 1 Department of Pharmacology, Dalhousie University, P.O. Box 15000, Halifax, NS B3H 4R2, Canada; elise.bisset@dal.ca; 2 Department of Medicine (Geriatric Medicine), Dalhousie University, P.O. Box 15000, Halifax, NS B3H 4R2, Canada

**Keywords:** healthspan, lifespan, vitamin supplements, frailty index, frailty phenotype, sex differences

## Abstract

Frailty is a state of accelerated aging that increases susceptibility to adverse health outcomes. Due to its high societal and personal costs, there is growing interest in discovering beneficial interventions to attenuate frailty. Many of these interventions involve the use of lifestyle modifications such as dietary supplements. Testing these interventions in pre-clinical models can facilitate our understanding of their impact on underlying mechanisms of frailty. We conducted a narrative review of studies that investigated the impact of dietary modifications on measures of frailty or overall health in rodent models. These interventions include vitamin supplements, dietary supplements, or amino acid restriction diets. We found that vitamins, amino acid restriction diets, and dietary supplements can have beneficial effects on frailty and other measures of overall health in rodent models. Mechanistic studies show that these effects are mediated by modifying one or more mechanisms underlying frailty, in particular effects on chronic inflammation. However, many interventions do not measure frailty directly and most do not investigate effects in both sexes, which limits their applicability. Examining dietary interventions in animal models allows for detailed investigation of underlying mechanisms involved in their beneficial effects. This may lead to more successful, translatable interventions to attenuate frailty.

## 1. Introduction

Daily consumption of dietary supplements and other dietary modifications are commonly proposed as a way to improve overall health. In Canada, 65% of women between 51 and 70 years of age use such supplements, but only 42% of men in the same age group do so [1]. While the use of supplements is widespread, there is less information regarding their effectiveness at improving health in older adults. One proposed use for supplements is to reduce frailty or attenuate the negative effects of age on health. Some clinical work has explored the effects of supplements on frailty, as reviewed recently [2]. The present narrative review will focus on studies that have evaluated the impact of nutritional supplements in pre-clinical (animal) models of aging, where overall health has been assessed or where frailty has been measured with a frailty assessment tool. Our goal is to highlight mechanisms responsible for the beneficial effects of these supplements on frailty and/or overall health. Specifically, we selected articles that used aging rodent models to explore the impact of dietary supplements and amino acid restriction on frailty itself or on other markers of healthspan. Where we have referred to clinical literature, we have done so to emphasise the translational nature of this research area (e.g., to provide background for the concept of frailty and to explain how various nutritional supplements are thought to impact human health).

## 2. Frailty

### 2.1. Definition of Frailty

While there is no single definition of frailty, the World Health Organization defines frailty as “a clinically recognisable state in which the ability of older people to cope with everyday or acute stressors is compromised by an increased vulnerability brought by age-associated declines in physiological reserve and function across multiple organ systems” [3]. It tends to be more prevalent in older individuals, with up to 25% of people over the age of 85 being considered frail [4]. Frailty is also more common in women than men [5], a trend that is also seen in pre-clinical models [6]. Due to the burden of frailty on health to the individual and wider society, there is a need to discover suitable interventions to attenuate frailty. While pharmaceutical therapies, such as enalapril [7] or rapamycin [8], or lifestyle changes such as exercise [9,10] show promising results, dietary interventions may also be beneficial in pre-clinical models of frailty.

### 2.2. Measuring Frailty

As with the lack of a universal definition of frailty, there is no single method to quantify frailty. The two most popular methods to measure frailty exemplify two different ways to conceptualise frailty. The first is the deficit accumulation model, or *frailty index*. This model considers frailty to be the result of the accumulation of sub-clinical deficits across multiple systems, which scale up and lead to clinically evident frailty. This model was initially proposed in clinical work [11], before being translated to a mouse model [12]. It has now been modified for use in rats [13], and dogs [14]. Each version of the index measures a set number of deficits across multiple systems and scores each one as absent, mild, or present. The sum of these deficits is divided by the total measured to give a ratio between 0 and 1. In mice, individual items (typically >30 items) can be scored as 0 (absent), 0.5 (mild), or 1 (severe) [15]. The higher the ratio, the frailer the individual.

The second main approach is known as the *frailty phenotype* and is based on physical frailty. The phenotype describes frailty as the depletion of physical reserves over time. This method was first developed for a clinical population [16], before being translated to mice [17]. The phenotype approach has also been applied to rats [18], primates [19], and dogs [20]. The frailty phenotype measures signs of physical weakness using several tests and scores the individual based on deviation from the group average. In mice, the frailty phenotype scores grip strength, walking speed, physical activity, and endurance [17]. A mouse fails each test if they score 1.5 standard deviations below the mean. A mouse is considered frail if they fail three or more tests, with a pre-frail mouse failing two tests [17]. Those that fail fewer than two tests are considered robust. Both the frailty index and the frailty phenotype have their strengths, as reviewed in detail elsewhere [21]. These methods to quantity frailty in pre-clinical models facilitate investigation into the underlying biology of frailty and allow testing of possible interventions.

### 2.3. Putative Frailty Mechanisms

As with aging, there is no single underlying pathway that induces frailty. Rather, frailty reflects disruption of normal homeostasis that results from changes due to both intrinsic and extrinsic factors. A review of the biology of frailty in pre-clinical models has recently been published [22], based on the seven pillars of aging [23]. In brief, the pillars of aging, and likely frailty, are macromolecular damage, epigenetic drift, disruption in proteostasis, metabolic dysregulation, dysregulated stress response, chronic inflammation, and stem cell exhaustion [23]. Frailty can result from disruption in one or more of these mechanisms that can be further exacerbated by environmental stressors. These so-called pillars of frailty have been targeted by dietary interventions with the goal of reducing overall frailty.

Of the proposed pillars of aging and frailty, many of the nutritional supplements reviewed here target changes in chronic inflammation. This age- and frailty-related increase in chronic inflammation, known as “inflammaging”, is manifest as an increase in pro-inflammatory markers and a decrease in the ability to deal with external stressors [24]. This can be seen clinically as dysregulation in serum cytokines, where frail individuals have higher levels of pro-inflammatory cytokines than those who are not frail [25]. Higher levels of circulating cytokines have also been associated with frailty in aging mice [26]. Multiple complex pathways are involved in inflammaging, and sex-specific differences have been reported in older individuals, with men having higher levels of proinflammatory cytokines than women [27]. Although less is known about links between inflammation and frailty, studies in aging mice reveal that levels of proinflammatory cytokines increase as frailty increases, but different cytokines correlate with frailty in males and females [26]. Thus, targeting inflammaging with dietary interventions has been proposed to reduce age-related pathologies [28] and may decrease frailty.

## 3. Dietary Interventions

The dietary interventions discussed here include vitamin supplements, dietary supplements, and amino acid restriction diets. Vitamin and dietary supplements differ from traditional pharmaceuticals as they have different regulations involved in assessing their efficacy and safety [29]. They are generally found in foods, and some of the supplements discussed here are similar to those that have been used in clinical frailty research [2]. This review will not focus on caloric-restriction diets in pre-clinical models, as this topic has been recently reviewed in detail elsewhere [30]. There is also some controversy regarding the ability to translate caloric restriction diets to humans [31].

### 3.1. Vitamin Supplements

As shown in Table 1, many of the interventions designed to attenuate frailty in pre-clinical models use vitamins. Vitamins are biologically active compounds that are important for health and that may or may not be partially synthesised endogenously. While all vitamins are crucial, only a few have been tested as frailty interventions, as discussed below.

#### 3.1.1. Vitamin D

As shown in Table 1, many studies have used vitamins as an intervention to attenuate frailty. The most common intervention is vitamin D_3_ (25-hydroxyvitamin D). Vitamin D_3_ is a prohormone formed in skin by the combination of ultra-violet light and a cholesterol derivative [32]. Interestingly, vitamin D_3_ is not readily found in food and must instead be synthesised. This makes vitamin D_3_ more like a hormone than a traditional vitamin [32]. Vitamin D_3_ has multiple physiological functions, such as maintaining skeletal muscle health [33], increasing bone density [34], and preserving cardiovascular health [35]. This is not surprising, as vitamin D receptors (VDRs) are found throughout the body [36]. The absence of vitamin D_3_ has also been linked to multiple pathologies. VDR knockout (KO) mice have higher mortality, lower weight, increased alopecia, and bone malformations when compared to wildtype controls [37]. VDR KO mice also tend to develop secondary hyperparathyroidism even when fed a high calcium, high phosphorus rescue diet [38]. Another important function of vitamin D_3_ is its role in maintaining calcium and phosphate homeostasis [32]. It regulates calcium absorption in the gut and controls serum levels of calcium [39]. Interestingly, similar pathological phenotypes occur in VDR KO mice, even when they are fed a high calcium rescue diet [40]. Another genetic mouse model of low vitamin D_3_ involves the hepatic CYP2R1 enzyme, which converts vitamin D_3_ into circulating 25-hydroxyvitamin D (25-OHD) [41]. CYP2R1 KO mice have enlarged livers, and very low circulating levels of calcium and phosphorus [41]. Interestingly, levels of the enzyme CYP2R1 decrease with age naturally in mice, leading to low levels of 25-OHD [42]. This suggests that aging mice may greatly benefit from vitamin D_3_ supplementation. These multi-system effects of vitamin D_3_, along with aging pathologies linked to vitamin D_3_ deficiency, make it a prime target as an intervention to mitigate frailty.

The importance of vitamin D_3_ in skeletal muscle health suggested to some researchers that it might reduce physical frailty. Studies in mice show that chronic vitamin D_3_ deficiency reduces skeletal muscle contractility [43], and more recent work shows that skeletal muscle metabolism is disrupted in VDR KO mice [44]. The use of vitamin D_3_ to improve physical health was investigated by Seldeen et al. [45] when young male mice (6 months old) were given diets either deficient in vitamin D_3_ (125 IU) or with sufficient levels of vitamin D_3_ (1000 IU) for 12 months [45], as summarised in Table 1. Mouse health was assessed by several physical performance measures (grip strength, balance, endurance, and time to exhaustion), but the physical phenotype was not assessed. Mice deficient in vitamin D_3_ had lower uphill sprint exhaustion times, reduced stride length and grip endurance but no change in grip strength [45]. These changes in physical performance were associated with an increased expression of genes that code for muscle atrophy pathways in the quadriceps [45]. However, there are no changes in serum markers of inflammation in these vitamin D_3_ deficient animals [45]. A follow-up study by the same group used older male mice (24 months old) and measured frailty with the frailty phenotype [46]. Instead of studying vitamin D_3_ insufficiency alone, they added another group with a high vitamin D_3_ diet (8000 IU). After the 4-month exposure period, mice with both insufficient and normal levels of vitamin D_3_ had higher frailty [46]. Importantly, this was not seen in the high vitamin D_3_ group [46]. Interestingly, they noted no increase in bone mineral density as might have been expected with high levels of vitamin D_3_ supplementation. Similarly, Liu et al. [47] measured frailty using a modified frailty index in middle-aged male rats (13 months) fed a vitamin D_3_ supplemented (1.8 IU/kg) diet for 8 months [47]. Rats that took vitamin D_3_ had significantly lower frailty index scores than their age-matched controls [47]. Unlike the work by Seldeen et al. [46], they did find a protective effect of vitamin D_3_ on bone mineral density in older rats [47]. The difference in results of vitamin D_3_ supplementation on bone mineral density may be due to the use of different doses (8000 IU vs. 1.8 IU/kg), varying timeframes (4 vs. 7 months), or differences in species (mouse vs. rat). Taken together, these studies indicate that vitamin D_3_ supplementation is a promising intervention to mitigate frailty, even if it is started later in life. This also highlights the importance of having sufficient vitamin D_3_ levels, as a lack of this essential nutrient may increase frailty. Importantly, these studies used only male rodents, which limits the applicability of this work. Future work should determine whether vitamin D_3_ supplements at similar doses and delivered over similar time frames are effective in older females. As there is still controversy on the precise mechanisms through which vitamin D_3_ exerts these beneficial effects, more work in this area is warranted.

#### 3.1.2. Vitamin C

Vitamin C or ascorbic acid is an essential vitamin that is obtained through the diet. It is absorbed through food and cannot be synthesised by humans. This makes vitamin C, unlike vitamin D, a true vitamin. Physiologically, vitamin C acts in a similar fashion to antioxidants and it is necessary for human health [48]. Vitamin C supplementation has been suggested to augment immune function either via antioxidant protection or by directly enhancing immune cell function [49]. For example, influenza virus A infected male mice show lower expression of proinflammatory cytokines in the lung when they are vitamin C deficient when compared to infected mice with adequate vitamin C levels [50]. By contrast, this result is not found in female mice [50]. There is also evidence that high doses of vitamin C kills cancer cells in mice [51] and that supplementation with this essential nutrient extends lifespan in murine models [52]. Combined, these studies suggest that vitamin C has the potential to affect frailty, especially via beneficial effects on the immune system. A complication related to vitamin C supplementation in mouse models is that, unlike humans, mice synthesise their own vitamin C [48]. Hence, many researchers use a Gulo KO model where the gulo enzyme (L-gulo-y-lactone oxidase), essential for vitamin C synthesis, is knocked out [53]. These mice have lower body weights, a significantly reduced lifespan, and higher serum cholesterol levels [53,54]. These findings suggest that increased levels of vitamin C may improve health by attenuating multiple underlying frailty mechanisms such as those involving inflammation.

Animal studies have not yet explored vitamin C as an intervention for frailty, although some studies show promising effects on both lifespan and overall markers of health, as shown in Table 1. To better investigate vitamin C’s antioxidant effects, Selman et al. [55] used female mice exposed to cold stress to increase oxidation. Young wildtype mice were kept in cold conditions (7 °C) and then administered lifelong vitamin C supplementation [55]. They found no improvement in energy expenditure, metabolism, or lifespan in cold-exposed mice fed vitamin C. Interestingly, this study also found that cold exposure alone had no effect on mouse lifespan, unlike previous work that has shown a decrease in lifespan when oxidation levels are increased [56]. Thus, these findings suggest that cold-induced oxidation may not be an ideal oxidation model [55]. Uchio et al. [57] used senescence marker protein 30 knockout (SMP30 KO) male mice to test this intervention. These SMP30 KO mice show increased tissue susceptibility to damage [58] and cannot produce vitamin C [59]. SMP30 KO mice were given either high or regular doses of vitamin C for 2 months before half the mice in each group were given dexamethasone as a glucocorticoid analog to mimic an increase in stress [57]. Mice fed high levels of vitamin C had preserved immune function, normal cytokine levels and preserved T-cell count after dexamethasone treatment [57]. This shows that vitamin C supplementation can maintain immune system function under stress. Thus, these studies show mixed results regarding the beneficial effects of vitamin C supplementation, with preservation of immune function in aging being the best characterised. Interestingly, while the study utilising male mice showed beneficial results [57], the one using females did not [55], suggesting possible sex-specific effects of vitamin C supplementation. Considering the detrimental effects of systemic immune dysfunction with age, future work could focus on vitamin C supplementation and its impact on inflammaging and frailty in both sexes.

#### 3.1.3. Vitamin E

Vitamin E, or α-tocopherol, is an essential vitamin which is mainly found in animal fats and plant oils. It is generally categorised as an antioxidant. Like other supplements, vitamin E has numerous physiological effects. For example, there is evidence that vitamin E can alter cytokine production in human and animal models [60]. Vitamin E is also implicated in neurological development, as young mice fed a vitamin E deficient diet have reduced cognition and increased brain oxidation [61]. This was further examined using α-tocopherol transfer protein (TTP) knockout mice. TTP plays a role in controlling systemic levels of vitamin E. Adult male and female mice without the TTP protein show inhibition of neurogenesis and increased expression of neurodegeneration genes along with increased signs of anxiety [62]. This suggests the importance of sufficient vitamin E, particularly in maintaining neurological health, which may translate to protection against age-related cognitive decline and potentially also attenuate the degree of frailty.

The impact of vitamin E supplements on frailty have not been fully investigated, but effects on lifespan and physical performance have been explored (Table 1). Focusing on antioxidant effects, Navarro et al. [63] fed mice a lifelong vitamin E supplementation diet. Interestingly, they found a sex-specific effect on survival, where males fed vitamin E had lower mortality, but this was not seen in females [63]. Using only the male mice, they determined that vitamin E supplementation improved motor coordination and exploratory behavior compared to controls. As in previous work, they found that males given vitamin E had less oxidative damage in their brains compared to controls [63]. This suggests that many of the health benefits of vitamin E may be mediated through protection against oxidation; however, future work is required, especially as these beneficial effects may not occur in females.

#### 3.1.4. Nicotinamide

Nicotinamide is the amide form of vitamin B_3_ and is a key component in the nicotinamide adenine dinucleotide pathway (NAD+). This compound can be both obtained from the diet and endogenously synthesised [64]. Interestingly, NAD+ levels decrease with age and this is linked to cellular senescence [65]. Many other aging processes including DNA damage, cognitive impairment, and mitochondrial changes are linked to lower NAD+ [66]. These are highlighted in an NAD+ deficient mouse model, C57Bl/6RccHsd, which has a nicotinamide nucleotide transhydrogenase gene deletion. Male C57Bl/6RccHsd mice exhibit a reduction in insulin sensitivity and altered metabolism compared to controls [67]. However, there are sex differences in the NAD+ pathway, where female mice are resistant to the metabolic dysfunction resulting from a nicotinamide deficiency unlike males [68]. These beneficial effects are promising as healthspan interventions and suggest that nicotinamide may be a useful intervention to reduce frailty [69].

The effects of nicotinamide supplementation on overall markers of health in pre-clinical models have been investigated, but the effects on frailty directly have not been measured (Table 1). Mitchell et al. [70] explored the beneficial effects of nicotinamide on metabolism. They fed 12-month-old male mice nicotinamide supplements with or without a high fat diet to induce obesity for their remaining life [70]. Neither of these diets resulted in a change in lifespan, but mice fed a high fat diet had improved locomotor activity when nicotinamide was also consumed [70]. This suggests that nicotinamide can offset some of the negative changes that occur with obesity in older male mice. However, when male mice are injected with nicotinamide supplements for 8 weeks, they develop insulin resistance and increased lipid accumulation in their skeletal muscle [71]. One reason for these differing results may be the use of different doses of nicotinamide (0.5 g/g and 1.0 g/kg in food vs. 100 mg/kg injected respectively). Beneficial effects were observed with lower doses while detrimental effects occurred at the higher doses, so the concentration-dependence of these effects should be further investigated. In addition, both studies used only male mice so future work should explore the effects of nicotinamide supplementation in females as well.

**Table 1:** Vitamin interventions to improve health and decrease frailty.

Intervention ^1^	Strain/ Species	Sex	Age (Mos)	Intervention	Health Assessment	Main Results	References
**Vitamin D_3_**	C57Bl/6 mice	Male	6	125 IU or 1000 IU of vitamin D_3_ for 12 months	Physical performance	Insufficient levels of vitamin D_3_ impair physical performance. Vitamin D_3_ has no effect on inflammatory markers.	[45]
	Fischer 344 rats	Male	6 or 13	0.045 µ/kg vitamin D_3_ for 7 months	27 item frailty index	Vitamin D_3_ Supplementation attenuates frailty and loss of bone mineral density with age.	[47]
	C57Bl/6 mice	Male	24	125 IU, 1000 IU or 8000 IU vitamin D_3_ for 4 months	Frailty phenotype	Mice with insufficient vitamin D_3_ levels have impaired physical performance and higher frailty scores, unlike mice with increased levels of vitamin D. There are no changes in BMD and few changes in body composition.	[46]
**Vitamin C**	Cold stressed C57Bl/6 mice	Female	4	180 mg/kg vitamin C for 18 months	Oxidative damage and lifespan	No change in lifespan, energy expenditure, metabolism, or measures of oxidative damage in mice given vitamin C.	[55]
	SMP30-KO and C57BL/6 mice	Male	0.5	20 mg/kg or 200 mg/kg vitamin C for 2 months	Immune function	High vitamin C lowers splenic atrophy damage caused by dexamethasone, unlike low vitamin C. Mice given high vitamin maintained immune function, measured as cytokine levels and T-cell proliferation, that was damaged by dexamethasone.	[57]
**Vitamin E**	CD1/UCadiz	Both	7	5.0 g/kg of vitamin E for lifelong	Lifespan, physical and neurological performance	Vitamin E increases lifespan in male but not female mice. In males only, vitamin E improves motor coordination, exploration activity and mitochondrial metabolism.	[63]
**Nicotinamide**	C57Bl/6 mice	Male	12	0.5 g/kg nicotinamide with high or low-fat diet for 15.5 months	Lifespan and metabolism	Nicotinamide reduces negative effect of a high fat diet Supplementation improves motor coordination without changes to lifespan	[70]

^1^ Vitamin interventions to mitigate frailty and improve overall markers of health. Mos = months; BMD = bone mineral density; IU = international unit.

### 3.2. Non-Vitamin Supplements

As shown in Table 2, many other non-vitamin dietary supplements have been utilised to increase lifespan, improve overall health and to attenuate frailty. These supplements vary from amino acid supplements to elemental metalloids, and organic molecules, as discussed in more detail below3.2.1. Allicin

#### 3.2.1. Allicin

Allicin is an organosulfur compound commonly found in garlic. While allicin was first investigated as a potential antibacterial agent, more recent studies have shown its potential as an antihypertensive agent [72]. It also has both anti-inflammatory and anti-tumour properties [72]. In addition, it acts as an antioxidant by reacting with thiol-possessing enzymes [72]. Consequently, garlic extracts have been proposed as anti-aging treatments for some time [73]. However, allicin has poor stability, making in vivo studies complex [74]. Like many other organic compounds, allicin impacts multiple biological pathways, and many of these are not well understood. To date only one pre-clinical study has focused on the effects of allicin on frailty. Liu et al. [47] administered low or high doses of allicin to middle-aged male rats (13 months) and followed them for 8 months [47]. They found that allicin attenuated the development of frailty over the intervention time frame, as measured by a frailty index tool [47]. High doses of allicin also increased bone mineral density and bone strength [47]. While the mechanisms underlying these beneficial effects of allicin are still poorly understood, it does show promising results in reducing frailty. Understanding the underlying mechanisms is an important next step. Future research should also incorporate female models, as no studies have yet investigated the effects of allicin on frailty in females.

#### 3.2.2. Glycine

Glycine is a nonessential amino acid. While it can be synthesised by most mammals and birds, it can also be metabolised from components in many foods. While it is normally considered a nonessential amino acid, chronically low glycine levels can lead to multiple pathologies, so it is generally considered to be a conditionally essential amino acid [75]. Indeed, chronic glycine deficiency can lead to metabolic disorders such as obesity and insulin resistance [76]. Interestingly, glycine levels decline with age, but this occurs only in men [77]. Glycine also declines with age in *Caenorhabditis elegans,* and glycine supplementation increases lifespan in this organism [78]. This work also showed that glycine supplementation can maintain the methionine cycle [78]. Indeed, one small study suggested that glycine supplementation increases lifespan in male rats by reducing methionine toxicity [79]. The methionine pathway is a combination of methionine metabolism and methyltransferases that methylate multiple substrates such as DNA, histones, and telomerase. Disruption in this cycle is a proposed mechanism of aging and frailty [22]. Together, this work shows that glycine can act as an anti-aging compound in *C. elegans* and additional studies in mammalian models would be of interest.

It is not yet clear whether glycine modulates frailty in pre-clinical models (Table 2). Miller et al. [80] gave both male and female mice lifelong glycine supplementation. They found that glycine extends lifespan and reduces body weight in females but not males [80]. Another study showed that a combination of glycine and the dietary supplement N-acetylcysteine improved lifespan and markers of overall health [81], as discussed in more detail in the next section. This work suggests that glycine supplementation may improve overall health, but potential mechanisms are not well understood and frailty itself has not been measured. It is also not clear whether rodents, like humans, have sex-specific changes in endogenous glycine levels with age. Future work should link changes in the methionine cycle and frailty and should determine if glycine levels change with age in both sexes.

#### 3.2.3. *N*-Acetylcysteine

*N*-acetylcysteine (NAC) is a L-cysteine precursor that is used as a dietary supplement, although it is also an approved pharmaceutical agent. NAC was approved by the Food and Drug Administration to treat acetaminophen toxicity. However, NAC is a naturally occurring plant antioxidant that is also sold as a supplement [82]. As a therapeutic agent, NAC acts as a precursor to glutathione and can help treat hepatic toxicity [83]. However, there are other suggested therapeutic uses for NAC. It has been suggested and/or approved for other conditions including bronchopulmonary disorders and cardiovascular disease [82]. NAC has also shown potential in extending lifespan in studies in *C. elegans* and *Drosophila* [84,85]. Thus, NAC has been suggested as an anti-aging therapy and is thought to act on multiple aging mechanisms.

While NAC has not been tested as an intervention to mitigate frailty, it does improve overall markers of health (Table 2). NAC was initially tested for its anti-aging abilities in the genetically heterogeneous mouse strain known as UM-HET3. This strain was developed by Flurkey et al. [86] to introduce a heterogeneous mouse strain, instead of the more commonly used highly inbred mouse strains, for aging research [86]. With this new mouse model, they tested lifelong NAC supplementation and found that NAC extended lifespan, but only in males. NAC did, however, reduce weight in both sexes compared to controls [86]. As mentioned earlier, NAC has also been combined with glycine (GlyNAC) and given to mice as a supplement [81]. Kumar et al. [81] administered a lifelong GlyNAC diet to adult (65 weeks old) male and female mice. They found a significant increase in longevity (23.7%) in both sexes [81]. This diet also protected them against the mitochondrial damage, nutrient sensing dysfunction, and genomic damage observed in older mice [81]. These findings suggest that the combination of GlyNAC may be even more beneficial at improving overall health compared to either NAC or glycine alone. It would be interesting to know whether these interventions can attenuate frailty.

#### 3.2.4. Alpha-Ketoglutarate

Alpha-ketoglutarate (AKG) is key compound in the Krebs cycle and therefore is important to overall metabolism and energy production. It helps determine the rate of the citric acid cycle and acts as a precursor for the amino acids glutamate and glutamine. AKG can be produced endogenously but its levels naturally decline with age [87]. This led Chin and colleagues [88] to investigate AKG supplementation in *C. elegans*. They found that AKG extended lifespan by about 50% and prevented the age-related phenotype of rapid, uncoordinated movement characteristic of older *C. elegans* [88]. They showed that AKG targeted aging mechanisms including the inhibition of ATP synthase and mTOR kinase inactivation [88]. This connection between extended lifespan, mTOR inhibition and AKG was further demonstrated in *Drosophila* where supplemented flies lived longer [89]. The mTOR pathway is a highly conserved pathway that is involved in nutrient sensing, apoptosis, and cell proliferation as well as other functions. This pathway is also involved in regulating innate immunity, and so it functions as an immunosuppressant, and it is the target of the anti-aging drug rapamycin [90]. As rapamycin can reduce frailty [91] through inhibition of mTOR, AKG may also be beneficial to attenuate frailty.

AKG has been directly studied as an intervention to reduce frailty in a recent pre-clinical study (Table 2). Asadi et al. [92] gave middle-aged (18-month-old) male and female mice an AKG salt supplement diet for their remaining life and measured frailty using a frailty index instrument [92]. They found that while only female mice exhibited an extended lifespan, AKG decreased the amount of time animals spent frail for both sexes [92]. They also noted that AKG played a role in chronic immune regulation by reducing plasma cytokine levels, but this change was more obvious in female mice [92]. While this is only a single study, it does show that AKG is a potential beneficial intervention in the setting of frailty.

#### 3.2.5. Selenium

Selenium is a trace-essential metalloid that is involved in forming selenoproteins, where the metalloid binds to cysteine residues. These selenoproteins have many physiological functions, including involvement in anti-inflammatory activities, thyroxine synthesis, and antioxidant activities [93]. In humans, selenium levels have been closely correlated with human aging [94], where daily selenium intake is positively correlated with longevity [95]. It seems likely that selenium exerts its beneficial effects through its antioxidant actions [94]. Selenium-deficient mice show higher protein turnover along with increases in glucose, which suggests that selenium plays an important role in metabolism [96]. These multiple physiological functions and anti-aging effects suggest that selenium may be beneficial as an intervention to improve health and reduce frailty.

The impact of selenium on overall health has been investigated in several studies (Table 2). Yang et al. [97] tested the effects of organic and inorganic selenium supplementation on fertility in middle-aged (12 months old) female mice supplemented for 8 weeks [97]. Selenium supplementation reduces the rate of apoptosis in ovarian tissue and improves antioxidant function [97]. Another study focused on selenium supplementation and physical activity/performance in older mice. Aging mice exposed to selenium showed higher normalised grip strength than control, along with improved skeletal muscle calcium homeostasis [98]. Recently Plummer et al. [99] fed selenium supplements to male mice for 16 weeks and found that selenium supplementation protected mice against diet-induced obesity and reduced insulin-like growth factor 1 levels in both males and females [99]. There is also evidence that selenium affects neurogenesis. Leiter et al. [100] determined that dietary selenium mimics the exercise induced increase in the selenium transporter and that this can reverse age-related cognitive decline [100]. It is possible that other trace-essential metalloids may improve overall health in aging. For example, although the effects of zinc alone have not been investigated in rodent models, a diet low in vitamins, selenium, and zinc reduces muscle force production in aging mice [101]. Taken together, these studies show the potential benefits of selenium supplementation in the context of aging. Future studies should focus on whether selenium can also improve overall healthspan by attenuating frailty.

#### 3.2.6. Resveratrol

Resveratrol is a polyphenol stilbenoid produced by many different plants, including grapes, as a natural anti-parasitic. In mammals, it acts as a potent antioxidant [102] and sirtuin activator. It was proposed as an anti-aging supplement after a seminal paper described its anti-cancer properties in 1997 [103]. Since then, resveratrol has been widely studied for its anti-aging and other potential health benefits. In terms of effects on lifespan, a library of the sirtuin family of NAD+-dependent protein deacetylase activators were used in budding yeast *S. cerevisiae* to determine if they could alter lifespan [104]. This study showed that resveratrol extends lifespan by activating sirtuin 1 (SIRT1) [104]. However, these anti-aging effects are species and strain specific, and resveratrol has more effect in worms and yeast than in rodents [105]. It is also possible that instead of being a direct SIRT1 activator, resveratrol activates AMPK which in turn activates SIRT1 [106]. Other than its anti-aging properties, resveratrol may attenuate multiple age-related pathologies. It acts to restore immune system function by activating the nuclear factor kappa beta/N-terminal kinase pathway [107]. Resveratrol also reduces cardiovascular disease [108,109] and cancer [110]. These multiple beneficial effects of resveratrol and its potential as an anti-aging agent suggest that resveratrol may also reduce frailty.

Resveratrol has been used as an intervention to assess its impact on frailty and other markers of health (Table 2). Kane and colleagues [111] administered resveratrol (100 mg/kg) to older male mice starting at 18 months of age and measured frailty with a frailty index. They showed that mice that received resveratrol had lower frailty scores when compared to age-matched controls [111]. Kan and colleagues [112] assessed exercise performance in resveratrol-treated (25 mg/kg) middle-aged male mice. Although resveratrol did not increase forelimb grip strength or swim times, it did reduce blood lactate levels and increased liver glycogen levels after exercise [112]. A similar study by Rodríguez-Bies and colleagues administered resveratrol (16–17 mg/kg) to old male mice to assess impacts on exercise capacity. Resveratrol reduces lipid biogenesis in skeletal muscles and increases mitochondrial biogenesis but has little effect on exercise capacity [113]. By contrast, others have reported that older male mice supplemented with resveratrol (15 mg/kg) showed increased time to exhaustion with better recovery after exercise [114]. Interestingly, these studies also found synergistic health benefits when combining aerobic exercise and resveratrol supplementation. This suggests that combining dietary supplements with other modifications such as exercise may be a highly effective strategy to mitigate frailty.

**Table 2:** Other supplement interventions to improve overall health and attenuate frailty.

Intervention ^1^	Strain/ Species	Sex	Age (Mos)	Intervention	Health Assessment	Main Results	References
**Allicin**	Fischer 344 rats	Male	6 and 13	4 mg/kg, 8 mg/kg, and 16 mg/kg allicin for 8 months	27-item frailty index	Allicin attenuates the development of frailty in old rats. Higher doses of allicin maintain bone mineral density in aged rats.	[47]
**Glycine**	UM-HET3 mice	Both	9	8% glycine for lifelong	Lifespan and body composition	Glycine results in small but significant increases in mouse lifespan. In female mice, glycine reduces body weight.	[80]
**N-acetylcysteine**	HET3 mice	Both	7	5 g/L or 10 g/L of N-acetylcysteine for lifelong	Lifespan and body composition	In males, both doses of N-acetylcysteine increase lifespan. This is not seen in female mice. Supplementation reduces body weight in both sexes.	[86]
**GlyNAC**	C57Bl/6 mice	Both	14.9	1.6 mg/g glycine and 1.6 mg/g N-acetylcysteine for lifelong	Lifespan and metabolism	Combined glycine and N-acetylcysteine increase mouse lifespan. The combined intervention protects mice against mitochondrial dysfunction and age-related changes in metabolism.	[81]
**Alpha-ketoglutarate**	C57Bl/6 mice	Both	18	2% per weight alpha-ketoglutarate for lifelong	Lifespan and frailty index	Alpha-ketoglutarate increases lifespan in female but not male mice and decreases the amount of time mice spend frail. Supplementation decreases serum cytokines in male but not female mice. There are no changes in senescent markers.	[92]
**Selenium**	ICR mice	Female	12	0.15 mg/kg or 0.33 mg/kg sodium selenite or selenium yeast for 6 weeks	Reproductive physiology	Selenium supplementation improves antioxidant capacity in old mice. Supplementation reduces the rate of apoptosis in ovarian tissue.	[97]
	C57Bl/6 mice	Both	20	5 mg/kg nanoSelenium food for 2 months	Physical performance	Selenium supplementation increases grip strength and increases maximal muscle twitch force	[98]
	C57Bl/6 mice	Both	2	0.0073% sodium selenite or 0.0037% selenomethionine for 4 months	Body composition, serum hormones	Selenium lowers adiposity and body weight Mice exposed to selenium have lower serum IGF-1, leptin, glucose, and adiponectin	[99]
	C57Bl/6 mice	Female	12 or 18	50nM seleno-L-methionine for 28 days or 4 weeks	Neurogenesis, and memory tests	Mice given selenium have increased signs of neurogenesis Mice exposed to selenium perform better at cognitive testing tasks compared to control mice	[100]
**Resveratrol**	C57Bl/6 mice	Male	18	100 mg/kg for 6 months	Frailty Index	Resveratrol reduces frailty relative to control mice	[111]
	C57Bl/6 mice	Male	16	25 mg/kg for 28 days	Physical performance	Resveratrol did not improve grip strength or endurance swim times After exercise, resveratrol fed mice had lower blood lactate levels, lower blood ammonia levels, and higher levels of liver glycogen	[112]
	C57Bl/6J mice	Male	2, 12 or 18	16–17 mg/kg for 4.5 months	Physical performance	Resveratrol did not improve time to exhaustion running on a treadmill Resveratrol improved latency to fall on a rotarod in old animals Resveratrol reduced lipid peroxidation levels and mitochondrial biogenesis in skeletal muscle	[113]
	Mice	Male	12 or 18	15 mg/kg for 4 weeks	Physical performance	Resveratrol improved swimming time to exhaustion in older mice Resveratrol reduced blood lactate levels, serum free fatty acids, and muscle lipid peroxidation, and increased mitochondrial biogenesis	[114]

^1^ Supplements other than vitamins that help improve markers of health and reduce frailty. Mos = months; IGF-1 = insulin-like growth factor 1; GlyNAC = mixture of glycine and n-acetylcysteine.

### 3.3. Amino Acid Restriction Diets

An additional approach to dietary modification is to remove compounds that are typically found in the diet. Although there are many restriction diets, this section will focus on amino acid restriction diets as shown in Table 3. It is important to note that while the diets reviewed here remove specific substances from the diet, they do not alter total caloric intake.

#### 3.3.1. Methionine Restriction Diets

Methionine restriction (MR) has been used to mimic traditional dietary restriction, by allowing for many anti-aging benefits without reducing total caloric intake [115]. As an anti-aging therapy, MR induces longevity in rats [116]. This study used male rats fed a lifelong 0.17% methionine diet, which is calculated as a percent weight of the total feed, starting from 4–6 weeks of age [116]. By contrast, control mice consumed a traditional 0.85% methionine diet [116]. This seminal study showing that MR increased lifespan led to several additional studies exploring mechanisms underlying the benefits of MR. As there are many recent reviews on the anti-aging pathways targeting MR [115,117,118], this will not be discussed in detail here. These anti-aging effects provide some evidence that MR may improve overall health and frailty.

MR diets have been well characterised for their metabolic impacts and effects on longevity. Recently they have also been utilised as an intervention to reduce frailty and improve overall health as shown in Table 3. Bárcena et al. [119] used the Hutchinson–Gilford progeria syndrome (HGPS) mouse models, which have a mutation that results in an accelerated aging phenotype [119,120]. These mice were fed a lifelong 0.12% MR diet. This resulted in increased lifespan, protection against loss of bone density, and protection against cardiovascular dysfunction when compared to mice fed a control diet [119]. These researchers noted that MR acts through multiple pathways to reduce the complications of HGPS including enhanced DNA repair, reduced inflammation, and beneficial metabolic changes [119]. Preliminary work by Mitchell et al. [121] showed that an MR intervention (0.1% MR) started late in life reduced frailty index scores in both male and female mice. Building on this preliminary work, Schultz et al. [122] used older male mice fed an MR diet (0.1% MR) for 6 months [122]. They used two frailty clocks that produce scores related to lifespan and healthspan. These are the FRIGHT (Frailty Inferred Geriatric Health Timeline) score, which calculates estimated biological age and the AFRAID (Analysis of Frailty and Death) score, which predicts time until mortality [122]. Interestingly, the MR diet reduces FRIGHT scores, increases AFRAID scores, and reduces frailty as measured with a frailty index [122]. These studies suggest that MR attenuates frailty and improves overall health of mice in both sexes even when it is introduced later in life. It will be interesting to explore the mechanisms underlying these beneficial effects of MR on frailty.

#### 3.3.2. Branched-Chain Amino Acid Restriction

Branched-chain amino acid (BCAA) restriction focuses on removing specific amino acids from the diet, such as leucine, valine, and isoleucine. The idea for this diet was based on studies that correlated low protein diets with better metabolic health [123]. While some groups focused on total protein reduction, others focused on specific amino acids, such as MR. Due to links between amino acid restriction and metabolism, most BCAA restriction interventions have focused on alterations in metabolism as a health outcome [124]. For example, when Zucker fatty rats, a model of metabolic syndrome, were fed a BCAA restriction diet they had lower levels of triglyceride storage in their hearts caused by a shift in substrate utilisation [125]. Because frailty is linked to impaired metabolism, the shift away from fat deposition suggests that BCAA restriction may help to improve select metabolic determinants of frailty.

Only one study has determined the effects of BCAA restriction on frailty. Richardson et al. [126] used the accelerated aging HGPS model (discussed above) compared to wildtype controls. Lifelong BCAA restriction increases lifespan in both male and female HGPS mice. Similarly, when BCAA restriction is initiated early in life for wildtype mice, it increases lifespan and attenuates frailty but only in male mice [126]. Conversely, when BCAA restriction was initiated at 16 months of age, there was no effect on lifespan in either sex, though it did attenuate the progression of frailty in both sexes [126]. In terms of underlying mechanisms, they found a decline in the mTOR pathway associated with BCAA restriction [126]. These results indicate that BCAA restriction may attenuate frailty, but more work should be done to determine the optimal age of intervention and explore mechanisms involved in the sex differences observed in these studies.

**Table 3:** Amino acid restriction interventions to enhance overall health and reduce frailty.

Intervention ^1^	Strain/Species	Sex	Age (Mos)	Intervention	Health Assessment	Main Results	References
**Methionine restriction**	HGPS mice	Both	1.5	0.12% methionine restriction, lifetime	Lifespan and metabolism	Methionine restriction increases lifespan in male and female aging-accelerated mice. This diet improves lipid profile and respiration rate in mice.	[119]
	C57Bl/6Nia mice	Male	21	0.1% methionine restriction for 6 months	Rockwood Frailty Index	Methionine restriction reduces frailty scores, along with calculated FRIGHT scores and increases AFRAID scores.	[122]
	C57Bl/6 mice	Both	21	0.1% methionine restriction for 6 months	Frailty Index	Methionine restriction reduces frailty in both sexes but increases lifespan in only male mice.	[121]
**Branched chain amino acid restriction**	C57Bl/6Nia mice	Both	16	Reduced amino acid diet or low-branched amino acid (BCAA) diet for life	26 item frailty index	BCAA restriction improves frailty but does not alter lifespan when started at 16 months A lifelong BCAA restricted diet improves frailty in males but not females	[126]

^1^ Amino acid restriction diets to improve health and reduce frailty. Mos = months; BCAA = branched chain amino acid; FRIGHT = Frailty Inferred Geriatric Health Timeline; AFRAID = Analysis of Frailty and Death.

## 4. Conclusions, Limitations and Future Directions

**Figure 1:**
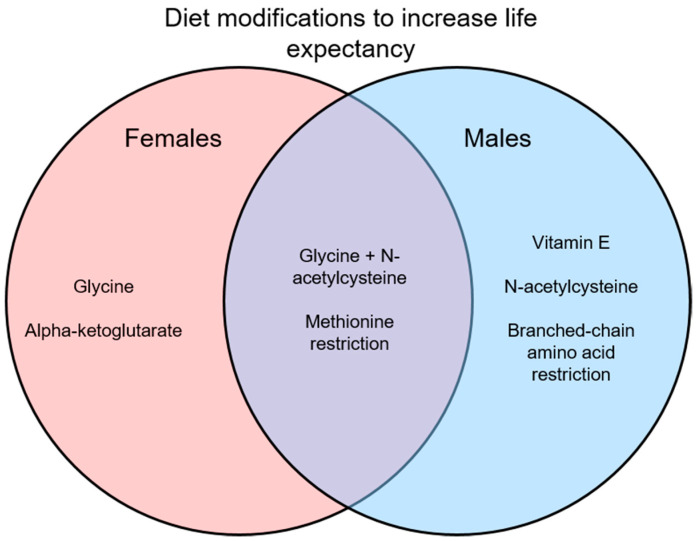
Interventions that increase life expectancy in males, females, or both.

**Figure 2:**
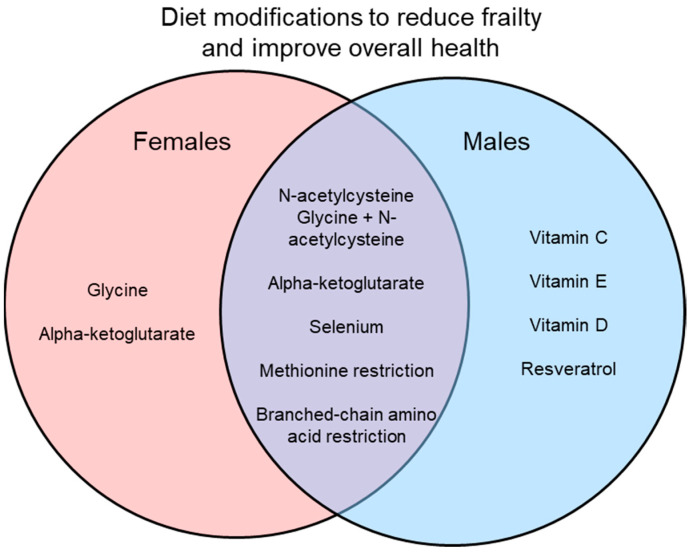
Interventions that improve markers of overall health and attenuate frailty in males, females, or both.

Many interventions have been suggested to reduce frailty and improve overall signs of health, with some of these interventions involving dietary modifications. Here, vitamin supplements, dietary supplements, and amino acid restriction diets in preclinical models have been reviewed as interventions for frailty. While many of these dietary changes do show positive results in improving health, most do not directly measure frailty or use only a single sex, mostly males. In studies that do use both sexes, they often find sex differences in the results as shown in Figure 1 and Figure 2. Interestingly, some interventions increase lifespan in a sex-specific manner (Figure 1), so studies of underlying mechanisms need to use both sexes as these also may differ in males and females. Similarly, studies of interventions on indices of overall health and frailty have found sex-specific effects (Figure 2). Another limitation is that while many studies did measure signs of health (i.e., metabolism, chronic inflammation), most did not measure frailty directly. It would be interesting to know how these markers of aging change in relation to dietary interventions and then link those changes to frailty scores.

This narrative review takes an in-depth look at specific studies of the impact of dietary supplements on overall health in aging rodent models and explores the underlying biology. We chose articles that used aging rodent models to investigate the effects of dietary supplements and amino acid restriction on frailty and other markers of healthspan. However, it is possible that there was some selection bias and that some articles were missed. Future work could include a systematic review and meta-analysis that aims to summarise all the work in this area. This may reveal additional interventions and their impact on aging and frailty in preclinical models. Our focus on pre-clinical models can be seen as both a limitation and a strength. While the goal of studying such interventions is eventual human translation, work in rodent models can allow the investigation of underlying mechanisms and a more controlled dietary environment. While there are some differences between rodents and humans in terms of responses to these interventions, many of the findings presented here are also seen in clinical studies [127,128]. This suggests that rodent models are useful to investigate and understand the impacts of currently used supplements on health. In the longer term, this promising pre-clinical work may pave the way for new approaches to help humans live heathier lives for longer.

Only results of lifespan studies that used both males and females are illustrated. Some interventions were effective in only one sex, which highlights the need to perform preclinical studies in males and females.

Results of only studies that used both males and females are illustrated. Some interventions improved healthspan in only one sex, while others were effective in both sexes. Preclinical studies that use both males and females are essential to explore the effects of interventions on healthspan and to investigate mechanisms underlying frailty.
